# Diverse Effects of Amino Acids on *Monascus* Pigments Biosynthesis in *Monascus purpureus*

**DOI:** 10.3389/fmicb.2022.951266

**Published:** 2022-07-15

**Authors:** Sheng Yin, Yiying Zhu, Bin Zhang, Baozhu Huang, Ru Jia

**Affiliations:** ^1^Beijing Advanced Innovation Center for Food Nutrition and Human Health, Beijing Technology and Business University, Beijing, China; ^2^Beijing Engineering and Technology Research Center of Food Additives, Beijing Technology and Business University, Beijing, China; ^3^School of Food and Health, Beijing Technology and Business University, Beijing, China

**Keywords:** amino acids, *Monascus* pigments, biosynthesis, *Monascus purpureus*, PKS cluster

## Abstract

Amino acids could act as nitrogen sources, amido group donors, or bioactive molecules in fungi fermentation, and consequently, play important roles in *Monascus* pigments (MPs) biosynthesis. But the understanding of the effects of various amino acids on MPs biosynthesis is still incomprehensive. In this work, 20 free amino acids were added to the fermentation medium to evaluate their effects on MPs biosynthesis in *Monascus purpureus* RP2. Six amino acids, namely, histidine (HIS), lysine (LYS), tyrosine (TYR), phenylalanine (PHE), methionine (MET), and cysteine (CYS), were selected as the valuable ones as they exerted significant effects on the production yield and even on the biosynthesis metabolic curves of MPs. Moreover, the dose-dependent and synergistic effects of valuable amino acids on MPs biosynthesis were observed by tests of serial concentrations and different combinations. In addition, it revealed that HIS and MET were the prominent amino acids with dominant and universal influences on MPs biosynthesis. The analog compounds of HIS (amitrole) and MET [calcium 2-hydroxy-4-(methylthio)] were added to the fermentation medium, and the results further confirmed the extraordinary effects of HIS and MET and their analogs on MPs biosynthesis. Furthermore, the gene transcription profile indicated that a differential expression pattern was observed in the polyketide synthase (PKS) cluster responsible for MPs biosynthesis in response to HIS and MET, revealing that they could oppositely regulate MPs biosynthesis in different ways. These findings would benefit the understanding of MPs biosynthesis regulation mechanism in *M. purpureus* and contribute to the industrial production of MPs by fermentation.

## Introduction

*Monascus* pigments (MPs) are a large class of secondary polyketide metabolites with a common azaphilone skeleton produced by *Monascus* spp. (Chen et al., 2015; Chen W. et al., 2017). Because of their bright red, orange, and yellow colors, MPs have been used as traditional natural colorants in China for thousands of years. The traditional natural colorant compounds are still widely used in industries of food, pharmaceutical, cosmetic manufacture, and dyeing in modern society (Lin et al., 2008; Mapari et al., 2010; Feng et al., 2012; Patakova, 2013; Dufosse et al., 2014; Mostafa and Abbady, 2014; Srianta et al., 2014; Chen et al., 2015). Consequently, continuous studies have been conducted on biosynthesis and the large-scale production of MPs.

Recent research advances reveal that MPs biosynthesis is controlled by the polyketide synthase (PKS) gene cluster. The PKS cluster is highly conserved in different *Monascus* species and is composed of a dozen of gene elements encoding various enzymes and transcription factors (Yang et al., 2014; Chen et al., 2015; Chen W. et al., 2017). However, due to the complex biosynthesis process involved with a dozen of biotransformation reactions, there are still controversies about the details on MPs formation pathway, especially regarding how the red, yellow, and orange pigments are dividedly synthesized. In addition, the expression and the regulation of the PKS gene cluster seem complicated and ambiguous.

It’s generally recognized that secondary metabolites biosynthesis in fungi is regulated by various factors, including temperature, pH, light, and carbon or nitrogen sources (Calvo et al., 2002; Calvo and Cary, 2015; Liu et al., 2016; Lind et al., 2018). For MPs biosynthesis in *Monascus* by fermentation, nitrogen sources are not only essential nutrients to support mycelium growth but also bioactive compounds that may exert regulatory effects. Previous reports indicated that the addition of different nitrogen sources in fermentation resulted in significant impacts on the yield and composition of MPs (Chen and Johns, 1993; Jung et al., 2003, 2005; Hajjaj et al., 2012; Zhang et al., 2013; Said et al., 2014; Shi et al., 2015). Jung et al. (2003) investigated the color characteristics and structures of the MPs derivatives produced by *Monascus* fermentation with separate addition of 20 amino acids. It revealed that the amino acids serine, glutamine, glycine, alanine, and histidine were responsible for red MPs production, and the amino acids phenylalanine, valine, leucine, and isoleucine contributed to yellow and orange MPs production. Besides, it’s confirmed that derivative MPs contained the moieties of some added amino acids, suggesting that some amino acids were incorporated into MPs. Hajjaj et al. (2012) investigated the effects of 13 free amino acids on red MPs production in *Monascus ruber* and found 5 amino acids (i.e., glycine, tyrosine, arginine, serine, and histidine) as sole nitrogen sources that favored red MPs production. Besides, *in vitro* chemical reactions demonstrated that some amino donors, such as arginine, lysine, γ-aminobutyric acid, and ammonia, directly get involved in the formation of red MPs molecules (Chen W. et al., 2017), suggesting that free amino acids could possibly be utilized as donors of amido group for MPs biosynthesis. Moreover, the specific bioactive amino acids, such as methionine and S-adenosylmethionine (SAM), play essential roles in multiple biological processes (Brosnan et al., 2007) and also get involved in MPs biosynthesis regulation (Yin et al., 2022).

Although some advances in the effects of amino acids on MPs biosynthesis have been achieved by previous studies, due to the complexity and diversity of the metabolism of different amino acids, systematic investigations are still indispensable to seek comprehensive insights into the roles of various amino acids in MPs biosynthesis and regulation. Therefore, this work investigated the effects of 20 free amino acids to select the valuable ones for MPs production in *Monascus purpureus* RP2, evaluated the dose-dependent and the synergistic effects of valuable amino acids, and conducted a comparative analysis of the different roles of histidine and methionine in PKS gene cluster expression and MPs biosynthesis. The findings in this study would benefit the understanding of MPs biosynthesis regulation in *M. purpureus* and contribute to the industrial production of MPs by fermentation.

## Materials and Methods

### Strains and Culture Conditions

The wild strain *M. purpureus* RP2 (Laboratory collection) was cultured at 30°C in potato dextrose agar (PDA) medium. For MPs production by liquid-state fermentation, *M. purpureus* RP2 was cultured at 30°C in PDA medium for 10 days, and the spore suspension was prepared by washing the mycelium with sterile water and collected by filtration with sterile gauze. The spore suspension (10%) was inoculated into seed medium (40 g/L rice flour, 8 g/L peptone, 5 g/L soybean meal, 1 g/L MgSO_4_⋅7H_2_O, 2 g/L KH_2_PO_4_, and 2 g/L NaNO_3_) and cultured at 33°C with vigorous shaking at 200 rpm on a shaking incubator with a rotational radius of 10 cm for 48 h. The seed culture (10%) was then inoculated into a fermentation medium (20 g/L glucose, 5 g/L yeast nitrogen base w/o amino acids, 5 g/L K_2_HPO_4_⋅3H_2_O, 0.5 g/L MgSO_4_⋅7H_2_O, 5 g/L KH_2_PO_4_, 0.1 g/L CaCl_2_, 0.03 g/L MnSO_4_⋅H_2_O, 0.01 g/L FeSO_4_⋅7H_2_O, and 0.01 g/L ZnSO_4_⋅7H_2_O) and cultured at 33°C with vigorous shaking at 200 rpm on a shaking incubator with a rotational radius of 10 cm for 14 days. For colony morphology observation, *M. purpureus* was cultured at 30°C in a fermentation medium containing agar (20 g/L) for 9 days.

### Effects of Addition of Amino Acids in Fermentation on *Monascus* Pigments Biosynthesis

A total of 20 amino acids (5 g/L) were added to the fermentation medium containing yeast nitrogen base without free amino acids, respectively, and liquid fermentation with *M. purpureus* RP2 was conducted for 14 days. The yield of red, yellow, and orange MPs was determined every 2 days during fermentation to investigate the influences of different amino acids on MPs biosynthesis in *M. purpureus* RP2.

The amino acids that exerted obvious effects on MPs biosynthesis were selected for further analysis of dose-dependent effect on MPs biosynthesis. Serial gradient concentrations (1∼9 g/L) of amino acids were added to the fermentation medium, and fermentation with *M. purpureus* RP2 and MPs production determination were performed as described earlier.

The synergistic effect of different amino acids on MPs biosynthesis was investigated by the combined addition of multiple amino acids that exhibited similar effects in the fermentation medium. Fermentation and MPs production determination were performed as described earlier.

To figure out the mode of action of specific amino acids, the selected amino acid was partially or completely replaced with its structural analog compound and added to the fermentation medium for fermentation and MPs production determination.

### Measurement of *Monascus* Pigments Production

For the measurement of MPs production, the fermentation broth was centrifuged at 8,000 × *g* for 10 min to collect mycelia and supernatant. The mycelia or broth supernatant samples were soaked in 50 ml of 70% (v/v) ethanol and incubated in the water bath (60°C) for 1 h to extract MPs. The mixture was centrifuged at 8,000 × *g* for 10 min to collect supernatant, which was used to measure MPs concentration by spectrophotometer (OD 410 nm for yellow MPs, OD 470 nm for orange MPs, and OD 505 nm for red MPs) (Yin et al., 2022).

### DNA Manipulation Techniques

Standard DNA manipulation techniques were performed as described by Green and Sambrook (2012). Total RNA from *M. purpureus* was prepared using the RNAprep Pure Plant Kit (TIANGEN, Beijing, China) following the manufacturer’s instructions. RNA was subjected to reverse transcription to generate cDNA using Quantscript RT Kit (TIANGEN, Beijing, China) following the manufacturer’s protocol. Quantitative Real-Time PCR (q RT-PCR) was performed using the SuperReal PreMix Plus (SYBR Green) Kit (TIANGEN, Beijing, China) in CFX96 Touch Real-Time PCR System (Bio-Rad, Hercules, CA, United States) with the following cycling conditions: 95°C for 2 min, followed by 40 cycles of 94°C for 20 s, 63°C for 45 s, and 60°C for 5 min. The *GAPDH* gene was used for transcript normalization. All reactions were performed in triplicate. Data were analyzed using the 2^–ΔΔCt^ method corrected for primer efficiencies using the untreated group mean as the reference condition (Schmittgen and Livak, 2008). Primers used for gene transcription level assay by q RT-PCR were listed in Supplementary Table 1.

### Comparative Analysis of *Monascus* Pigments Biosynthesis Polyketide Synthase Gene Cluster From *Monascus purpureus* Strains

The PKS gene cluster responsible for MPs biosynthesis in *M. purpureus* RP2 was analyzed by genome sequencing, assembly, and comparative analysis. The genome of *M. purpureus* RP2 was sequenced by single-molecule, real-time (SMRT) sequencing technology. Sequencing was performed at the Beijing Novogene Bioinformatics Technology Co., Ltd. The Augustus 2.7 program was used to retrieve the related coding gene. Databases used to predict gene functions included GO, KEGG, COG, NR, TCDB, Swiss-Prot, and TrEMBL. The secondary metabolite biosynthesis gene clusters were predicted with antiSMASH. A whole-genome Blast search was performed against the above databases. Genomic alignment between the sample genome and reference genome was performed using the MUMmer and LASTZ tools.

## Results and Discussion

### Effects of Different Amino Acids on *Monascus* Pigments Biosynthesis

**FIGURE 1:**
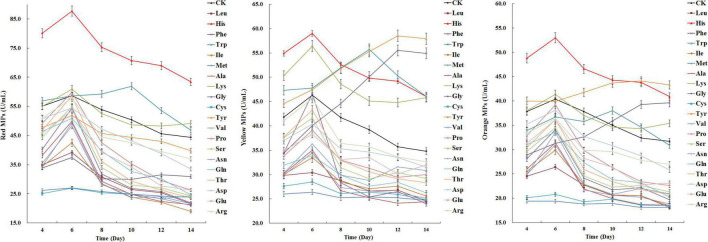
Effects of 20 amino acids on *Monascus* pigments (MPs) production in *Monascus purpureus* RP2 fermentation. CK, *M. purpureus* RP2 fermentation without the addition of amino acid; Leu, *M. purpureus* RP2 fermentation with the addition of leucine (LEU); His, *M. purpureus* RP2 fermentation with the addition of histidine (HIS); Phe, *M. purpureus* RP2 fermentation with the addition of phenylalanine (PHE); Trp, *M. purpureus* RP2 fermentation with the addition of tryptophan (TRP); Ile, *M. purpureus* RP2 fermentation with the addition of isoleucine (ILE); Met, *M. purpureus* RP2 fermentation with the addition of methionine (MET); Ala, *M. purpureus* RP2 fermentation with the addition of alanine (ALA); Lys, *M. purpureus* RP2 fermentation with the addition of lysine (LYS); Gly, *M. purpureus* RP2 fermentation with the addition of glycine (GLY); Cys, *M. purpureus* RP2 fermentation with the addition of cysteine (CYS); Tyr, *M. purpureus* RP2 fermentation with the addition of tyrosine (TYR); Val, *M. purpureus* RP2 fermentation with the addition of valine (VAL); Pro, *M. purpureus* RP2 fermentation with the addition of proline (PRO); Ser, *M. purpureus* RP2 fermentation with the addition of serine (SER); Asn, *M. purpureus* RP2 fermentation with the addition of asparagine (ASN); Gln, *M. purpureus* RP2 fermentation with the addition of glutamine (GLN); Thr, *M. purpureus* RP2 fermentation with the addition of threonine (THR); Asp, *M. purpureus* RP2 fermentation with the addition of aspartic acid (ASP); Glu, *M. purpureus* RP2 fermentation with the addition of glutamic acid (GLU); Arg, *M. purpureus* RP2 fermentation with the addition of arginine (ARG).

The effects of amino acids on MPs biosynthesis in *M. purpureus* RP2 were systematically investigated by fermentation with the addition of 20 free amino acids using the fermentation medium containing YNB without free amino acids as the nitrogen source. Fermentation results showed that various patterns of changes in MPs biosynthesis were observed in response to the addition of different amino acids (Figure 1). Compared with the fermentation without free amino acids, most amino acids added to the fermentation medium exhibited inhibiting effects to different degrees on MPs production, while histidine (HIS), lysine (LYS), tryptophan (TRY), tyrosine (TYR), and phenylalanine (PHE) gave rise to increased MPs production (Figure 1). HIS was the only amino acid that led to the most significant increase in all three kinds of MPs biosynthesis; the peak yields of red, yellow, and orange MPs were raised by 49, 27, and 31%, respectively. The addition of LYS only exerted a positive effect on yellow MPs biosynthesis, whose peak yield was increased by 22%. Besides, it’s noteworthy that TRY, TYR, and PHE not only promoted MPs production but also obviously changed the metabolic curve of MPs biosynthesis; the fermentation time when the peak yield of MPs appeared was postponed from 6 to 10 days and 12 days, which consequently facilitated more MPs generation. However, TYR and PHE only acted on yellow and orange MPs biosynthesis in the special pattern, and they reduced the production of red MPs and hardly affected the normal metabolic curve. With regard to the amino acids with negative influences on MPs biosynthesis, methionine (MET) and cysteine (CYS) were the most outstanding ones and contributed to the approximate 50% decrease in yields of red, yellow, and orange MPs.

### Dose-Dependent Effects of Different Amino Acids on *Monascus* Pigments Biosynthesis

**FIGURE 2:**
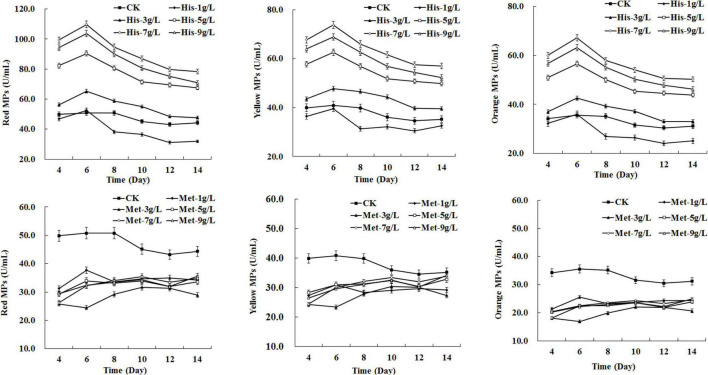
Effects of amino acids at different concentrations on *Monascus* pigments (MPs) production in *Monascus purpureus* RP2 fermentation. CK, *M. purpureus* RP2 fermentation without the addition of amino acid; His, *M. purpureus* RP2 fermentation with the addition of HIS; Met, *M. purpureus* RP2 fermentation with the addition of MET.

To investigate the effect of different concentrations of amino acids on MPs biosynthesis, serial gradient concentrations (1–9 g/L) of selected amino acids (HIS, LYS, TYR, PHE, MET, and CYS) were added to the fermentation medium, respectively. The results indicated that the positive or negative impact on MPs biosynthesis exerted by specific amino acids generally depended on the added dose. As shown in Figure 2, the production of red, yellow, and orange MPs synchronously increased as the concentration of HIS rose from 3 to 7 g/L, while 1 g/L of HIS decreased the MPs production and the yield of MPs generated by 9 g/L of HIS was lower than that of 7 g/L. The addition of LYS only enhanced yellow MPs biosynthesis at the concentration of 3 and 5 g/L, and 9 g/L of LYS extremely reduced all three kinds of MPs production by about 50∼60% (Supplementary Figure 1). TYR enhanced yellow and orange MPs biosynthesis at each concentration, and 3 g/L was detected as the optimal dose (Supplementary Figure 1). PHE led to a continuous sharp rise in yellow and orange MPs biosynthesis during 14 days of fermentation at a concentration of higher than 1 g/L, and a positive correlation was observed between MPs yield and PHE dose ranging from 3 to 7 g/L (Supplementary Figure 1). With respect to MET and CYS, each concentration from 1 to 9 g/L displayed an inhibiting effect on three kinds of MPs production, and the dose with significant effect was observed to be 3 g/L for MET and 5 g/L for CYS (Figure 2 and Supplementary Figure 1).

### Synergistic Effects of Different Amino Acids on *Monascus* Pigments Biosynthesis

**FIGURE 3:**
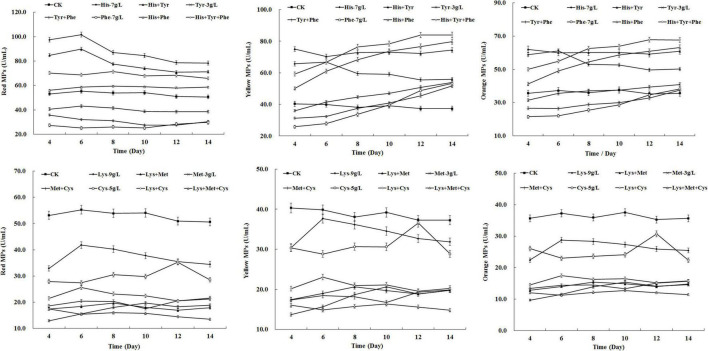
Effects of combination of amino acids on *Monascus* pigments (MPs) production in *Monascus purpureus* RP2 fermentation. CK, *M. purpureus* RP2 fermentation without the addition of amino acid; His, *M. purpureus* RP2 fermentation with the addition of HIS; Tyr, *M. purpureus* RP2 fermentation with the addition of TYR; Phe, *M. purpureus* RP2 fermentation with the addition of PHE; Met, *M. purpureus* RP2 fermentation with the addition of MET; Lys, *M. purpureus* RP2 fermentation with the addition of LYS; Cys, *M. purpureus* RP2 fermentation with the addition of CYS.

To investigate the synergistic effect of amino acids on MPs biosynthesis, the amino acids that displayed positive effects (7 g/L of HIS, 3 g/L of TYR, 7 g/L of PHE) and negative effects (3 g/L of MET, 5 g/L of CYS, 9 g/L of LYS) on MP production were mixed and added to the fermentation medium of *M. purpureus* RP2. As shown in Figure 3, HIS exerted a dominant role in enhancing the yields of red, yellow, and orange MPs. When mixed with TYR and PHE, HIS obviously reversed the negative effect of TYR and PHE on red MPs production and further promoted the positive effect of TYR and PHE on yellow and orange MPs production (Figure 3). TYR and PHE exhibited a dominant role in altering the typical biosynthesis metabolic curves of yellow and orange MPs, but they hardly gave a remarkable contribution to the yield (Figure 3). It revealed that HIS, TYR, and PHE displayed a synergistic enhancement effect on the biosynthesis of yellow and orange MPs. The synergistic effect was also observed among MET, CYS, and LYS, and their combination led to a maximum decrease in the yield of all three kinds of MPs in comparison with the addition of any one or two amino acids (Figure 3). But it’s indicated that MET was the one with the dominant influence on inhibiting MPs biosynthesis. Although different amino acids mentioned above expressed the synergistic promoting or inhibiting effect on MPs production, it’s noteworthy that HIS and MET were the dominant ones that exerted universal effects on MPs biosynthesis.

### Effects of Analogs of Histidine and Methionine on *Monascus* Pigments Biosynthesis

**FIGURE 4:**
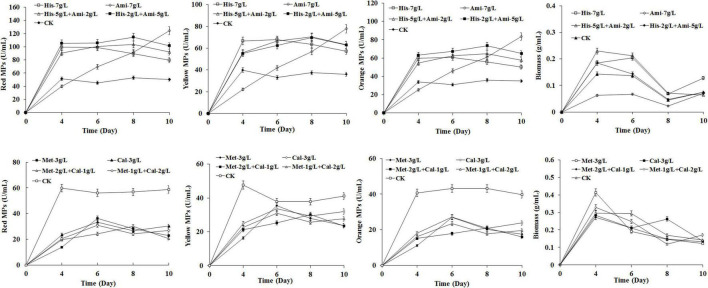
Effects of analogs of HIS and MET on *Monascus* pigments (MPs) production in *Monascus purpureus* RP2 fermentation. CK, *M. purpureus* RP2 fermentation without the addition of amino acid; His, *M. purpureus* RP2 fermentation with the addition of HIS; Ami, *M. purpureus* RP2 fermentation with the addition of amitrole; Met, *M. purpureus* RP2 fermentation with the addition of MET; Cal, *M. purpureus* RP2 fermentation with the addition of calcium 2-hydroxy-4-(methylthio) butyrate.

To investigate the mode of action of HIS and MET on MPs biosynthesis, the HIS analog amitrole (3-amino-1, 2, 4-triazole) and the MET analog calcium 2-hydroxy-4-(methylthio) butyrate (CHMTB) were used to completely or partially substitute for the two amino acids in the fermentation of *M. purpureus* RP2. As shown in Figure 4, CHMTB represented almost the same effect as MET on both MPs production and the biomass of *M. purpureus* RP2, revealing that it followed the metabolism mode similar to MET. With respect to amitrole, significant differences were detected in MPs biosynthesis and growth of *M. purpureus* RP2 between HIS and its analog. The mixture of amitrole and HIS at the total concentration of 7 g/L led to the increased yields of MPs, and the yields went higher as the proportion of amitrole rose in comparison with 7 g/L of HIS, but amitrole did not change the metabolic curve of MPs biosynthesis in the presence of HIS. However, in the absence of HIS, amitrole at 7 g/L totally changed the metabolic curve of MPs biosynthesis, which went straight up during fermentation, and more MPs were generated than that of HIS or the mixture after 9 days of fermentation. But 7 g/L of amitrole indeed inhibited the growth of *M. purpureus* RP2 since the biomass was quite lower than that of HIS or the mixture. The results suggested that the HIS analog amitrole probably was not incorporated into the HIS metabolism network in *M. purpureus* RP2, and consequently, it could continuously promote MPs biosynthesis and accumulation. With respect to the structural similarity with HIS, amitrole is the derivative of 1H-1, 2, 4-triazole substituted by an amino group (Supplementary Figure 2). It has toxicity in humans and is widely used as an herbicide. Heim and Larrinua (1989) revealed that amitrole toxicity in *Arabidopsis thaliana* was not caused by histidine starvation nor by the accumulation of a toxic intermediate of the histidine pathway, suggesting that this compound did not interfere with histidine metabolism. Hence, it is possible that amitrole could continuously contribute to MPs production due to its characteristics of structural similarity with HIS and no limitations from HIS metabolism regulation. When it comes to CHMTB, the analog shares a high structural similarity with MET except the amino group substituted by hydroxyl (Supplementary Figure 2). It is commonly used as an effective feed nutrient supplement of dietary MET for livestock (Lobley et al., 2006). This compound has the same biological potency as MET, and it proved that CHMTB could be converted to MET within body tissues (Lobley et al., 2006). Therefore, it is supposed that CHMTB added to the fermentation medium could be utilized *via* the MET metabolic pathway in *M. purpureus* RP2, which probably accounts for the same effects of MET and CHMTB on MPs biosynthesis.

### Histidine and Methionine Regulated *Monascus* Pigments Biosynthesis Genes Expressions in Different Ways

**FIGURE 5:**
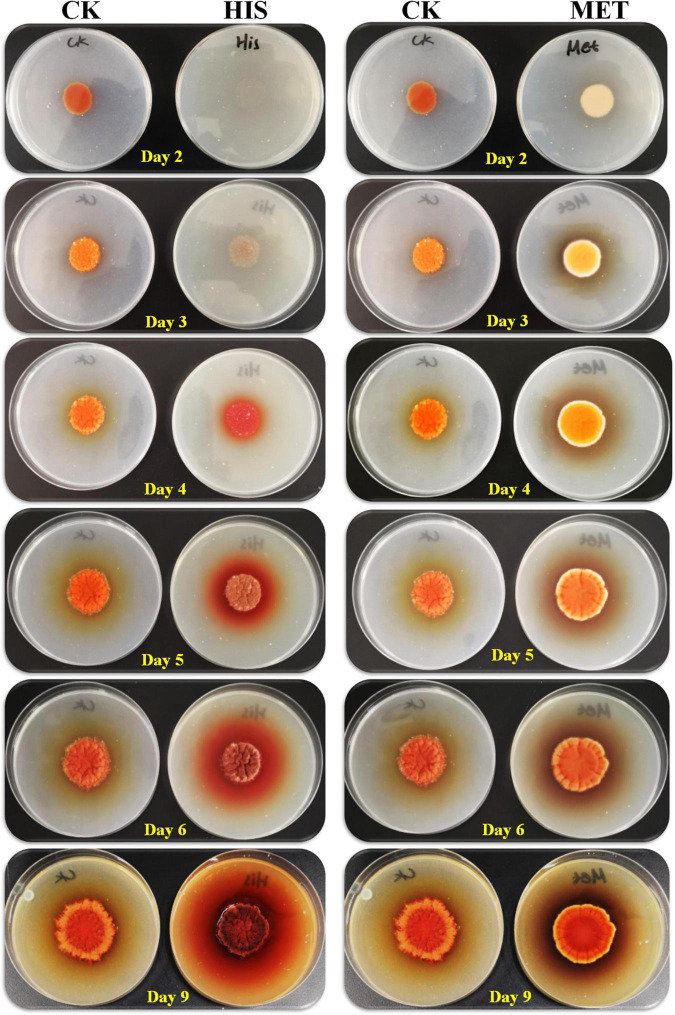
Growth of *Monascus purpureus* RP2 in fermentation plates with the addition of HIS and MET. CK, growth of *M. purpureus* RP2 in the fermentation plate without the addition of amino acid; HIS, growth of *M. purpureus* RP2 in the fermentation plate with the addition of HIS; MET, growth of *M. purpureus* RP2 in the fermentation plate with the addition of MET.

Secondary metabolites biosynthesis is coupled with asexual and sexual development in *Aspergillus* fungi (Lind et al., 2018). Hence, 7 g/L of HIS and 3 g/L of MET were added to the fermentation agar plates for the cultivation of *M. purpureus* RP2. Colony morphology observation indicated that significant differences were detected between colonies of *M. purpureus* RP2 cultured in plates with HIS and MET (Figure 5). The addition of MET contributed to the early colony development and consequent MPs production of *M. purpureus* RP2 after 2–3 days of cultivation, while the colony formation in the plate with HIS laid behind and the colony size was quite smaller than that of MET. Besides, the colony morphology in the plate with MET was quite different from that in the plate with HIS or without amino acid, indicating that the addition of MET changed the mycelial development of *M. purpureus* RP2. With respect to MPs yield, the addition of HIS generated much more MPs as the bright colors of red, orange, and yellow MPs became much more intensive within and around the colony during the later period of cultivation (Figure 5).

**FIGURE 6:**
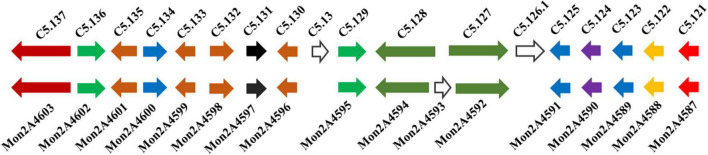
Polyketide synthase (PKS) gene clusters from *Monascus purpureus* YY-1 (C5.121-5.137) and *M. purpureus* RP2 (Mon2A 4587-4603). C5.121 and Mon2A4587 (100 and 98% identity with GenBank No. ANS12245.1): MFS multidrug transporter. C5.122 and Mon2A4588 (100% identity with GenBank No. ANS12244.1): decarboxylase. C5.123 and Mon2A4589 (100% identity with GenBank No. QGA67179.1): deacetylase. C5.124 and Mon2A4590 (100% identity with GenBank No. QGA67182.1): monooxygenase. C5.125 and Mon2A4591 (100% identity with GenBank No. QGA67225.1): acyltransferase. C5.126.1 (100% identity with GenBank No. QGA67184.1): ankyrin repeat protein. C5.127 and Mon2A4592 (100% identity with GenBank No. QGA67211.1): fatty acid synthase β subunit. Mon2A4593 (100% identity with GenBank No. TQB69636.1): hypothetical protein. C5.128 and Mon2A4594 (100% identity with GenBank No. QGA67228.1): fatty acid synthase α subunit. C5.129 and Mon2A4595 (100% and 95% identity with GenBank No. QGA67229.1): transcription factor. C5.13 (100% identity with GenBank No. TQB67922.1): hypothetical protein. C5.130 and Mon2A4596 (100% identity with GenBank No. APZ73943.1): reductase like-protein. C5.131 and Mon2A4597 (100% identity with GenBank No. APZ73942.1): serine hydrolase. C5.132 and Mon2A4598 (100% identity with GenBank No. APZ73941.1): FAD dependent dehydrogenase. C5.133 and Mon2A4599 (100% identity with GenBank No. AHA93896.1): oxidoreductase. C5.134 and Mon2A4600 (100% identity with GenBank No. AGI63864.1): 3-*O*-acetyltransferase. C5.135 and Mon2A4601 (98 and 100% identity with GenBank No. AGI63865.1): short-chain alcohol dehydrogenase. C5.136 and Mon2A4602 (100 and 99% identity with GenBank No. QGA67219.1): transcription factor. C5.137 and Mon2A4603 (100 and 99% identity with GenBank No. QGA67237.1): polyketide synthase.

The classical PKS gene cluster is responsible for MPs biosynthesis in *Monascus*. To investigate the influences of HIS and MET on MPs biosynthesis genes expression, the PKS gene cluster was searched in *M. purpureus* RP2 and analyzed by genome sequencing. Analysis results showed that a conserved PKS cluster was found, and it consisted of 15 functional enzymes encoding genes and two regulatory elements (Figure 6). The PKS cluster shared high homologies in sequences of most genes and a high similarity in structure composition with that of *M. purpureus* YY-1 (Yang et al., 2014). Two unrelated or unknown genes (C5.126.1 and C5.13) present in the PKS cluster of *M. purpureus* YY-1 were found to be vanished from that of *M. purpureus* RP2 (Figure 6), suggesting that those genes were redundant for MPs biosynthesis and probably were abandoned during evolution. *M. purpureus* RP2 also harbored a redundant gene (*Mon2A4593*) encoding a hypothetical protein with unknown function, which was inserted between the fatty acid synthase α and β subunits genes (*Mon2A4592* and *Mon2A4594*) and fortunately did not lead to disruption of the fatty acid synthase.

**FIGURE 7:**
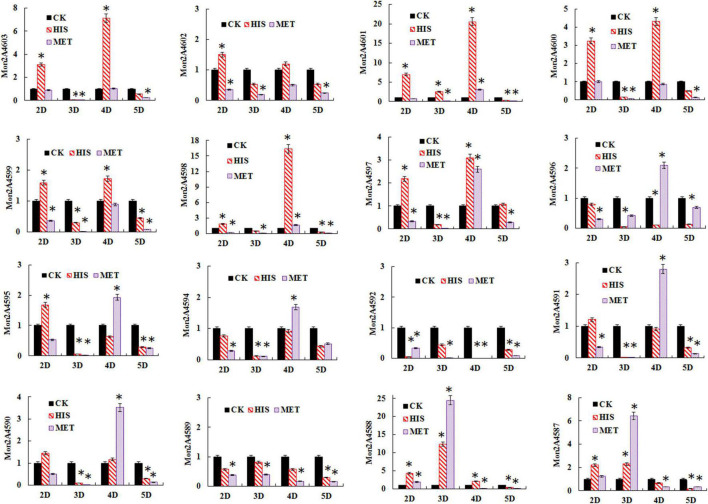
Gene transcription profile of the polyketide synthase (PKS) cluster in *Monascus purpureus* RP2 fermentation with HIS and MET. CK, *M. purpureus* RP2 fermentation without the addition of amino acid; HIS, *M. purpureus* RP2 fermentation with the addition of HIS; MET, *M. purpureus* RP2 fermentation with the addition of MET; D, fermentation time (Day). Bars with asterisks (*) are significantly different (*P* < 0.05).

**FIGURE 8:**
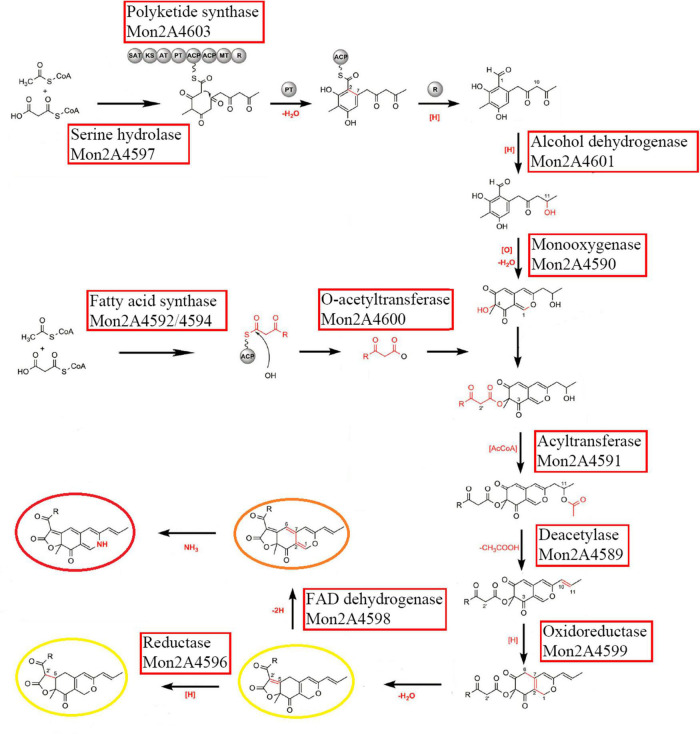
Putative *Monascus* pigments (MPs) biosynthesis pathway involved with the polyketide synthase (PKS) enzymes in *Monascus purpureus* RP2. The figure was modified with reference to Scheme 2 by Chen W. et al. (2017).

The transcriptional levels of the PKS cluster genes were further profiled in *M. purpureus* RP2 fermentation with HIS and MET. Differential expressions of the PKS genes were observed in response to HIS and MET (Figure 7). Generally, in comparison with fermentation without amino acid addition, HIS enhanced transcriptions of most of the PKS cluster genes, while MET generated the reversed results. According to previous reports on the MPs biosynthesis pathway (Chen W. et al., 2017; Liang et al., 2018), 12 key enzymes encoded by the PKS cluster probably got involved in MPs biosynthesis in *M. purpureus* RP2 (Figure 8). The expression levels of 6 key enzymes (PKS Mon2A4603, short-chain alcohol dehydrogenase Mon2A4601, 3-*O*-acetyltransferase Mon2A4600, oxidoreductase Mon2A4599, FAD-dependent dehydrogenase Mon2A4598, and serine hydrolase Mon2A4597) encoding genes were significantly increased as MPs biosynthesis enhanced in fermentation with HIS. Liang et al. (2018) reported that PKS (C5.137), short-chain alcohol dehydrogenase (C5.135), 3-*O*-acetyltransferase (C5.134), reductase like-protein (C5.130), and fatty acid synthase (C5.127 and C5.128) exhibited high expression levels when pigment production rapidly increased in *M. purpureus* YY-1 after 8 days of cultivation. Although the results shared a basic consistency, expressions of reductase-like protein (Mon2A4596), fatty acid synthase (Mon2A4594 and Mon2A4592), and deacetylase (Mon2A4589) were downregulated by the addition of HIS and MET in *M. purpureus* RP2. With respect to the two regulatory elements in the PKS cluster, it’s reported that they were expressed at higher levels under high pigment production conditions in *M. purpureus* YY-1 (Yang et al., 2014) and *M. purpureus* M9 (Chen D. et al., 2017). In *M. purpureus* RP2, expressions of Mon2A4602 and Mon2A4595 were both upregulated by HIS and downregulated by MET, which probably accounted for differential expressions of most PKS enzymes encoding genes and different MPs production yield in response to HIS and MET.

Even though the PKS cluster responsible for MPs biosynthesis is confirmed to be highly conserved in different *Monascus* strains, the temporal and spatial expression pattern of the PKS genes is still indistinct. Nevertheless, in the conditions of this work, most functional genes of the PKS cluster displayed an opposite expression pattern in response to the addition of HIS and MET, revealing that the two amino acids with universal and dominant influences could regulate MPs biosynthesis by controlling the PKS cluster expression. It is noteworthy that the addition of MET also led to a remarkable change in the colony morphology, while HIS hardly exerted the same effect. Since there is a close correlation between mycelial development and secondary metabolites biosynthesis in fungi (Lind et al., 2018), it is reasonable to speculate that MET could also regulate MPs biosynthesis by controlling the mycelial development. Regarding the molecular mechanism of MPs biosynthesis regulation by amino acids, it is believed that there are diverse pathways according to various metabolic networks of different amino acids. In our previous study (Yin et al., 2022), it was evident that MET inhibited MPs biosynthesis by inducing the expression of SAM synthetase; however, other regulation pathways possibly exist because a seemingly contradictory result was observed that the addition of SAM indeed promoted MPs biosynthesis. The gene expressions of several important enzymes (histidine decarboxylase, histidine methyltransferase, and aminotransferase) involved in HIS metabolism were also assayed, but no significant changes were found in response to the addition of HIS in the fermentation of *M. purpureus* RP2 (data not shown). Previous studies reported that histidine degradation and utilization played crucial roles in metal homeostasis management, nutritional flexibility, and virulence in fungi (Brunke et al., 2014; Dietl et al., 2016). In addition, histidine phosphorylation involved with histidine phosphotransferase and histidine kinase was proved to regulate cell growth, sporulation, secondary metabolism, morphological differentiation, biofilm formation, and virulence in fungi (Li et al., 2010; Fuhs and Hunter, 2017; Mohanan et al., 2017). These research progresses indicated that histidine could participate in metabolic regulation in different ways. As for the regulatory role of HIS in MPs biosynthesis, it’s indispensable to dig out more detail information on the molecule components and their interactions involved in the regulation network.

Secondary metabolites biosynthesis in fungi is regulated by various factors, including temperature, pH, light, and carbon or nitrogen sources (Calvo et al., 2002; Calvo and Cary, 2015; Liu et al., 2016; Lind et al., 2018). In this work, we systematically investigated the effects of the addition of different amino acids in the fermentation on MPs biosynthesis in *M. purpureus* RP2. It revealed that specific amino acids played a role of regulatory factor rather than nitrogen molecule in MPs biosynthesis, which not only affected MPs production yield but also altered MPs biosynthesis metabolic curves. Although the molecular mechanism of MPs biosynthesis regulation by amino acids is unclear and needs necessary in-depth studies, the findings in this work would benefit the understanding of MPs biosynthesis regulation mechanism in *M. purpureus* and contribute to the industrial production of MPs by fermentation.

## Data Availability Statement

The datasets presented in this study can be found in online repositories. The names of the repository/repositories and accession number(s) can be found below: NCBI BioProject - PRJNA842148, SAMN28647902, JAMLEA000000000.

## Author Contributions

SY conceived and designed the experiments, wrote the manuscript, and contributed to manuscript revision, financial support, and supervision. YZ, BZ, BH, and RJ performed the experimental work and data analysis. All authors contributed to the article and approved the submitted version.

## Conflict of Interest

The authors declare that the research was conducted in the absence of any commercial or financial relationships that could be construed as a potential conflict of interest.

## Publisher’s Note

All claims expressed in this article are solely those of the authors and do not necessarily represent those of their affiliated organizations, or those of the publisher, the editors and the reviewers. Any product that may be evaluated in this article, or claim that may be made by its manufacturer, is not guaranteed or endorsed by the publisher.
