# Supplementary material for: Diverse Effects of Amino Acids on Monascus Pigments Biosynthesis in Monascus purpureus
Source: Front Microbiol. 2022 Jul 15;13:951266. doi: 10.3389/fmicb.2022.951266 (PMC9335072; doi:10.3389/fmicb.2022.951266)
Supplement: Supplementary file 1 [file Data_Sheet_1.PDF]

Supplementary Material

Supplementary Table

Table 1 Primers used in this work

Gene	Primer	Sequence (5'-3')
Mon2A4603	4603-F	AGATTACTGTCGTTGAAG
	4603-R	CTCTCAGGATACTCTCAT
Mon2A4602	4602-F	GTTCTGGATGGCGTATAG
	4602-R	ACCTGTTCGTTCAATGTC
Mon2A4601	4601-F	ATACCATTGTCTATCTCAC
	4601-R	GATTTCGTCTTTCATAGC
Mon2A4600	4600-F	TTGATGTCTTGATCTGTC
	4600-R	CTATATACTCCGCATACG
Mon2A4599	4599-F	TACTGGAACGATGCCTAC
	4599-R	CACTTCGGTCAGGTTATAC
Mon2A4598	4598-F	AATACAGCCTCCAGAAGA
	4598-R	TGTTCATCACCGTAATGG
Mon2A4597	4597-F	AAGACGAAGGATGTTGTG
	4597-R	TCAAATCCGTCTCTGGAG
Mon2A4596	4596-F	ATCATCTCGTTCAAGTTCAAG
	4596-R	GATGCCGTTGTGATTGAC
Mon2A4595	4595-F	TGTGTACTCTCTCCCTCTC
	4595-R	ATTCCGACGACATTGCTAT
Mon2A4594	4594-F	CTCCTGATTGAACTACTT
	4594-R	ATATCTCTGGATGGTCTC
Mon2A4592	4592-F	ATTATCAAGCGACTCAAT
	4592-R	TCAATGTATGTCATCTCAG
Mon2A4591	4591-F	AACTTCAATGAGGCATATTATG
	4591-R	TAGTTCCACGCTCAATTC
Mon2A4590	4590-F	AAGTCTATTGGCGTTCTC
	4590-R	GTCTACGTCTATAGTCTTCAG
Mon2A4589	4589-F	GAGAAGAACGTCCGCTTG
	4589-R	AACTCCAGCAGCAACTTC
Mon2A4588	4588-F	TACATTGCCAGTTTACAG

	4588-R	GTATGGAGGATTAGTTCAC
Mon2A4587	4587-F	TGGCATCTTACTATCTCC
	4587-R	GAATGTAGACTCCACTCA
GAPDH	GAPDH-F	CCGTATTGTCTTCCGTAAC
	GAPDH-R	GTGGGTGCTGTCATACTTG

Supplementary Figure

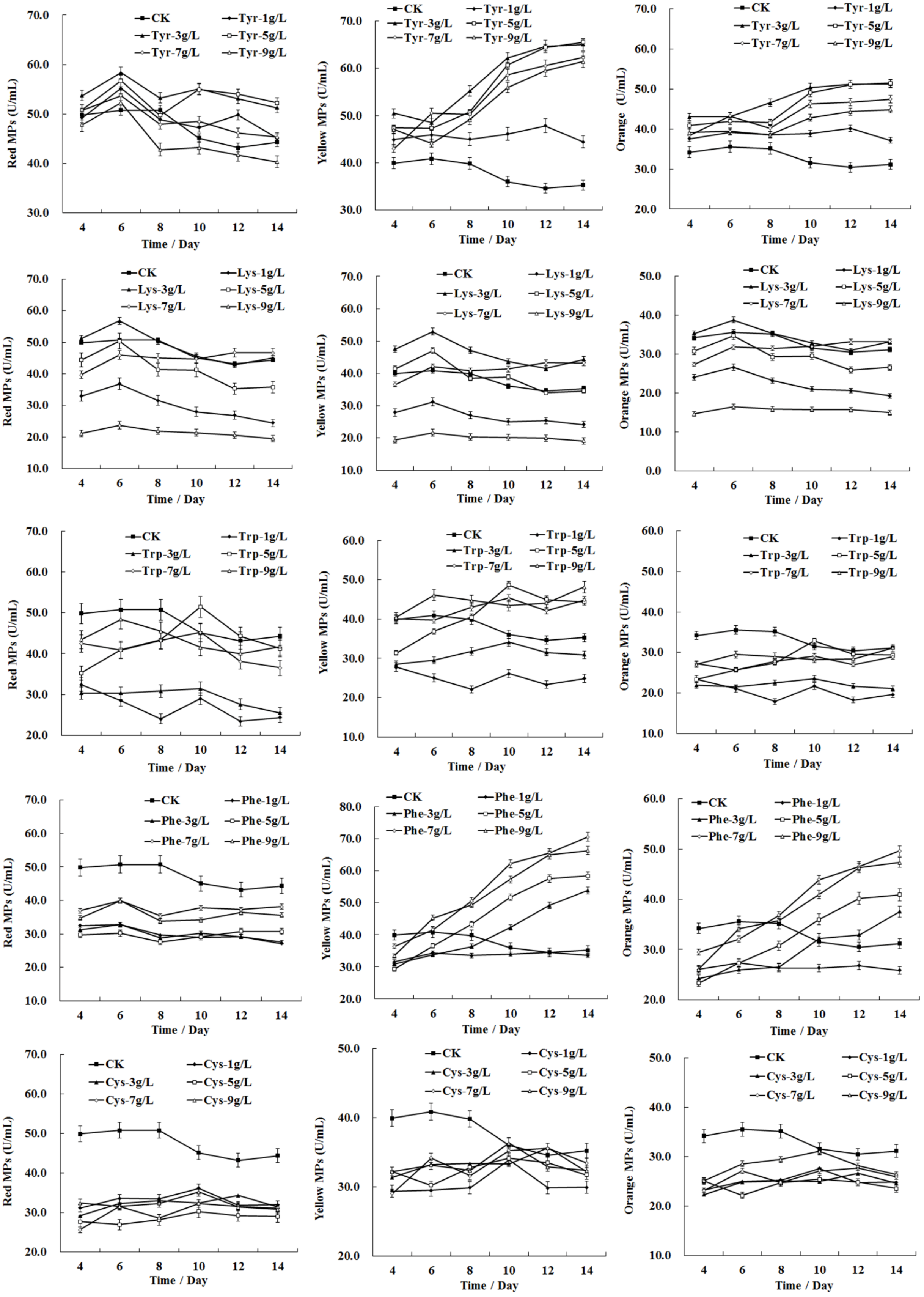

Supplementary Figure 1. Effect of amino acids at different concentrations on MPs production in *M. purpureus* RP2 fermentation. CK, fermentation without amino acids.

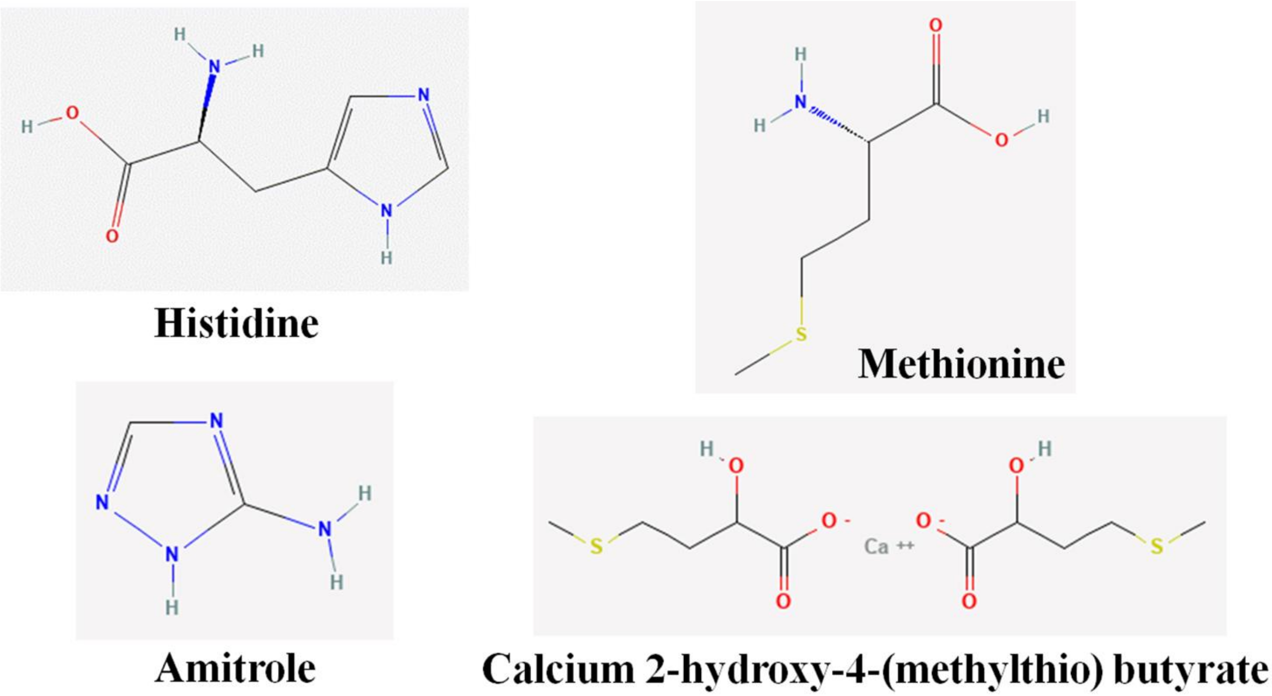

Supplementary Figure 2. Structure of histidine, amitrole, methionine, and calcium 2-hydroxy-4-(methylthio) butyrate.
